# Compromised reactive but intact proactive inhibitory motor control in Tourette disorder

**DOI:** 10.1038/s41598-022-05692-z

**Published:** 2022-02-09

**Authors:** Indrajeet Indrajeet, Cyril Atkinson-Clement, Yulia Worbe, Pierre Pouget, Supriya Ray

**Affiliations:** 1 Edmond and Lily Safra Center for Brain Sciences, The Hebrew University of Jerusalem, Jerusalem, Israel; 2 Centre of Behavioural and Cognitive Science, University of Allahabad, Prayagraj, India; 3 Sorbonne University, INSERM U1127, CNRS UMR7225, UM75, ICM, Movement Investigation and Therapeutics Team, Paris, France; 4 Department of Neurophysiology, Saint Antoine Hospital, Assistance Publique-Hôpitaux de Paris, Paris, France

**Keywords:** Neuroscience, Psychology, Diseases, Neurology

## Abstract

Tourette disorder (TD) is characterized by tics, which are sudden repetitive involuntary movements or vocalizations. Deficits in inhibitory control in TD patients remain inconclusive from the traditional method of estimating the ability to stop an impending action, which requires careful interpretation of a metric derived from race model. One possible explanation for these inconsistencies is that race model’s assumptions of independent and stochastic rise of GO and STOP process to a fixed threshold are often violated, making the classical metric to assess inhibitory control less robust. Here, we used a pair of metrics derived from a recent alternative model to address why stopping performance in TD is unaffected despite atypical neural circuitry. These new metrics distinguish between proactive and reactive inhibitory control and estimate them separately. When these metrics in adult TD group were contrasted with healthy controls (HC), we identified robust deficits in reactive control, but not in proactive control in TD. The TD group exhibited difficulty in slowing down the speed of movement preparation, which they rectified by their intact ability to postpone the movement.

## Introduction

The ability to regulate and/or inhibit unwanted movements is one of the prerequisites for flexible goal-directed behavior. For example, driving through a busy street warrants more alertness, precaution, and preparedness of the driver to react to sudden appearance of a pedestrian or vehicle on the collision course by pressing the brake to avoid an accident. The process of adjusting attentional setting to detect the critical signals (e.g., attentive driver) or strategic adjustment in ones’ response (driving at slow speed) to optimize success in goal-directed behavior (e.g., avoid accidents) is referred to as proactive control^1,2^. Whereas the process of outright withholding/cancelation of a planned unwanted movement (e.g., to withhold pressing the gas-paddle) when instructed is referred to as reactive inhibitory control^3,4^. The dual mechanisms framework of cognitive control associates proactive control with the maintenance of goal-relevant information and sustained attention in anticipation of upcoming event(s), and reactive control with transient late correction in response to the appearance of specific stimuli^5^. A successful inhibition of action is the outcome of the interplay between both proactive and reactive inhibitory control *(see the special issue by Giovanni Mirabella*^6^).

In laboratory, proactive and reactive inhibitory control are often assessed using tasks like Go/No-Go task, AX version of continuous performance task (AX-CPT), cued stop-signal task and classical countermanding task^7–9^. In a Go/No-Go task, the requirement for the elicitation (or inhibition) of a movement in a stipulated time is indicated at the beginning of each trial by a go (or no-go) signal. Infrequent presentation of the no-go signal and random interleaving of go and no-go trials make the task difficult. The frequency of errantly eliciting a response in no-go trials is referred to commission error rate, which is widely used as a metric of proactive inhibitory control^10^. Less the commission error, better the proactive inhibitory control. In contrast, participants generate a primary motor response following a go-signal in the majority of trials in a countermanding (or stop-signal) task, but they are instructed to cancel the pre-planned response in randomly interleaved trials when a stop-signal appears after a variable delay from the go-signal, called stop-signal delay. In ideal situation, error in inhibition increases monotonically with the increase in stop-signal delay. Race model^3^ describes performance in the stop-signal task as an outcome of a competition between a preparatory GO and an inhibitory STOP process. Each process independently rises at a stochastic rate to reach a common fixed threshold. If the STOP process reaches the threshold before the GO, the movement is inhibited; the movement is generated otherwise. Race model provides methods to estimate the average time taken by the STOP process called stop-signal response time (SSRT). SSRT is used as a metric of reactive inhibitory control. The shorter the SSRT, the better the reactive inhibitory control.

The average SSRT in patients population has been contrasted with healthy control (HC) to identify the propensity of impairment in inhibitory control in the populations with motor and cognitive deficits like Parkinson’s disease^11,12^, schizophrenia^13–15^, obsessive–compulsive disorder (OCD)^16,17^, attention-deficit hyperactivity disorder (ADHD)^18,19^, and Tourette disorder (TD)^20,21^. Tourette disorder (TD) represents a relevant model of inhibition impairment due to its major clinical sign: tics. Usually, they corresponded to sudden, repetitive, non-rhythmic, involuntary or semi-voluntary movements and/ or vocalization^22,23^ and are frequently considered as *“fragments of motor behavior that escape voluntary motor control”*, related to a deficiency in inhibitory control of actions^20,24,25^. However, studies did not find a consistent significant difference between TD and HC either in the commission error rates^17,26–28^ or in SSRT^20,29^. A recent study^30^ examined volitional (proactive and reactive) and automatic inhibition in primary tic disorder using conditional stop-signal and masked priming task respectively. They found that proactive control was intact and automatic inhibition was impaired. Though they found longer SSRT in the overall tic group than HC, it was associated with the presence of OCD. There was no significant difference in SSRT between tic without OCD group and HC. It led authors to speculate that inconsistent findings of difference in SSRT may be an outcome of no accounting for comorbidities in TD. Another possible explanation for the inconsistent findings of difference in SSRT is that SSRT might not be an appropriate measure of inhibitory control when the context independence assumption (i.e., STOP process does not influence GO process) is violated, especially at short SSDs^31,32^. The violation is pervasive and has a severe effect on the estimation of SSRT. For example, if trials with short intervals between the go and stop signal are removed from SSRT estimation, the difference reported in published studies between HC and disorder groups mostly disappears^33–35^.

The cancellable rise-to-threshold model (CRTT)^32,36^, which is an alternative to race model, does not assume a race between GO and STOP. In its original form, this model advocates that the inhibition of a pre-planned movement is determined by the efficiency of the detection process and magnitude of deceleration. After the stop-signal appears, deceleration in the build-up activity of GO is triggered. If the magnitude of deceleration determined by the detectability of the stop-signal is sufficient, the impending movement is inhibited. We and others^9,32,35^ have recently shown that cancellable rise-to-threshold model can explain the behavior in stop-signal task better than race model. To assess the contributions of both proactive and reactive control in movement inhibition without independence assumption, we derived a pair of metrics on the foundation of CRTT framework of countermanding, which we referred to as ‘proactive delay’ and ‘log-attenuation rate’, respectively^9,32,36^. The former estimates the time consumed in postponing the response in expectation of the stop-signal to increase the odds of successful inhibition, whereas the latter estimates the dynamics of decelerated preparatory build-up activity triggered by the onset of the stop-signal. Since CRTT metrics are independent of the race model’s assumptions and sensitive to deficits in proactive and reactive inhibitory control independently, we revisited the deficits in inhibitory control in adult TD patients^21^.

Previously we showed that the efficacy of inhibitory control depends on both deceleration in the speed of movement preparation in response to the stop signal, and strategic postponement of movement initiation^9^. In the current study we tried to characterize inhibitory control in TD in the CRTT framework. Given the deficiency in inhibitory control of actions in TD^20,24,25^, we expected that both the CRTT metrics, i.e. log-attenuation rate and proactive delay, would be less in TD in comparison to HC. However, we found that the TD group rectified the deficiency in reactive control by judicious exertion of intact proactive control.

## Results

**Figure 1:**
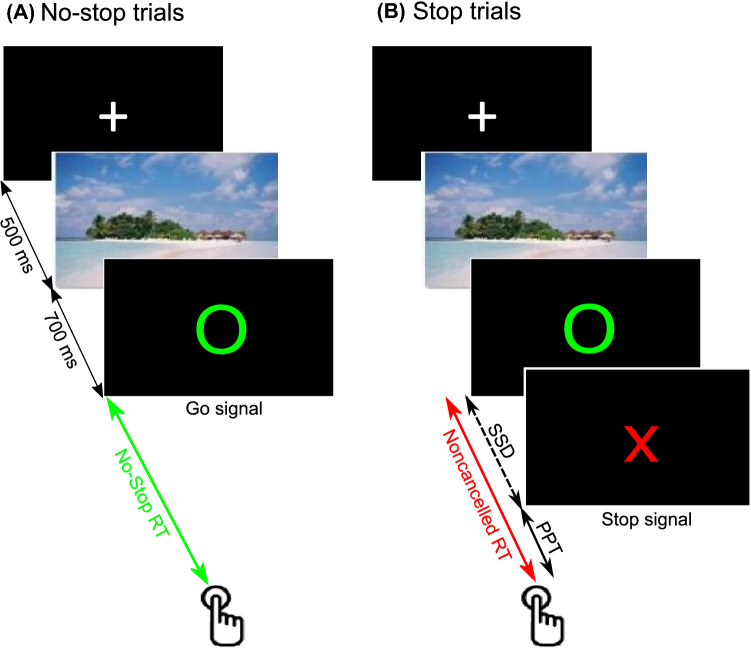
Schematic of Emotional Stop Signal Task (ESST). There were a total of 405 trials. Each trial began with the display of a fixation cross (+) in white color at the center of the display. After 500 ms, an image of a scene with emotional content appeared at the center for 700 ms. Subsequently, a blank display in black appeared for a variable 1–2 s interval. It was followed by a green unfilled circle at the center. (**A**) In the majority (66%) of trials (no-stop trial), participants were instructed to press the ENTER key on the keyboard “*as soon as possible*”. (**B**) In the rest of the trials (stop trials), after a variable delay from the circle, the letter X in capital in red color appeared at the center, instructing participants to refrain from pressing the ENTER key. No-stop and stop trials were pseudorandomly presented. The figure is taken from Atkinson-Clement et al., 2020 with permission^21^.

A total of 63 TD and 34 HC performed an Emotional Stop Signal Task (ESST), which required them to refrain from pressing a key in response to an infrequent stop-signal^21^ (see Fig. 1 and “Materials and methods”).

### Participant’s demographics

Table 1 shows the details of demographics and incidence of comorbidities. The data collected from 14 participants (10 TD and 4 HC) were removed from analyses due to incomplete behavioural data or misunderstood instructions. A total of 53 TD (Sex: 11 females, 42 males, mean ± SD age: 30.21 ± 10.5 years, education: 14.25 ± 2.56 years) and 30 HC (Sex: 09 females, 21 males, mean ± SD age: 31.63 ± 10.44 years, education: 14.57 ± 2.93 years) was available for this study. In this sample, there was no significant difference in age [*t* (81) = 0.596, *p* (two-tailed) = 0.553, *d* = 0.136, BF_01_ = 3.624]; education level [*t* (81) = 0.522, *p* (two-tailed) = 0.603, *d* = 0.119, BF_01_ = 3.756]; or gender [${\rm X}^{2}$(1, *N* = 83) = 0.895, *p* = 0.344, Cramer's *V* = 0.012] between the TD and HC groups. Out of 53 TD patients, 19 were taking antipsychotic medication, 23 had ADHD, and 11 had OCD. The mean (SD) tic severity was 16.321(7.076) as measured by YGTSS/50.

**Table 1:** Demographic and comorbidity details of participants. The table shows that there was no significant difference in gender ratio, age, or years of education between TD and HC.

	TD	HC	p	BF_01_
Number of participants	53	30	0.344	
Gender (F/M)	11/42	9/21		
Mean (SD) age (years)	30.21 (10.5)	31.63 (10.44)	0.553	3.624
Mean (SD) years of education	14.25 (2.56)	14.57 (2.93)	0.603	3.756
Medication	19	0		
ADHD	23	0		
OCD	11	0		

### Behavioural performances

**Figure 2:**
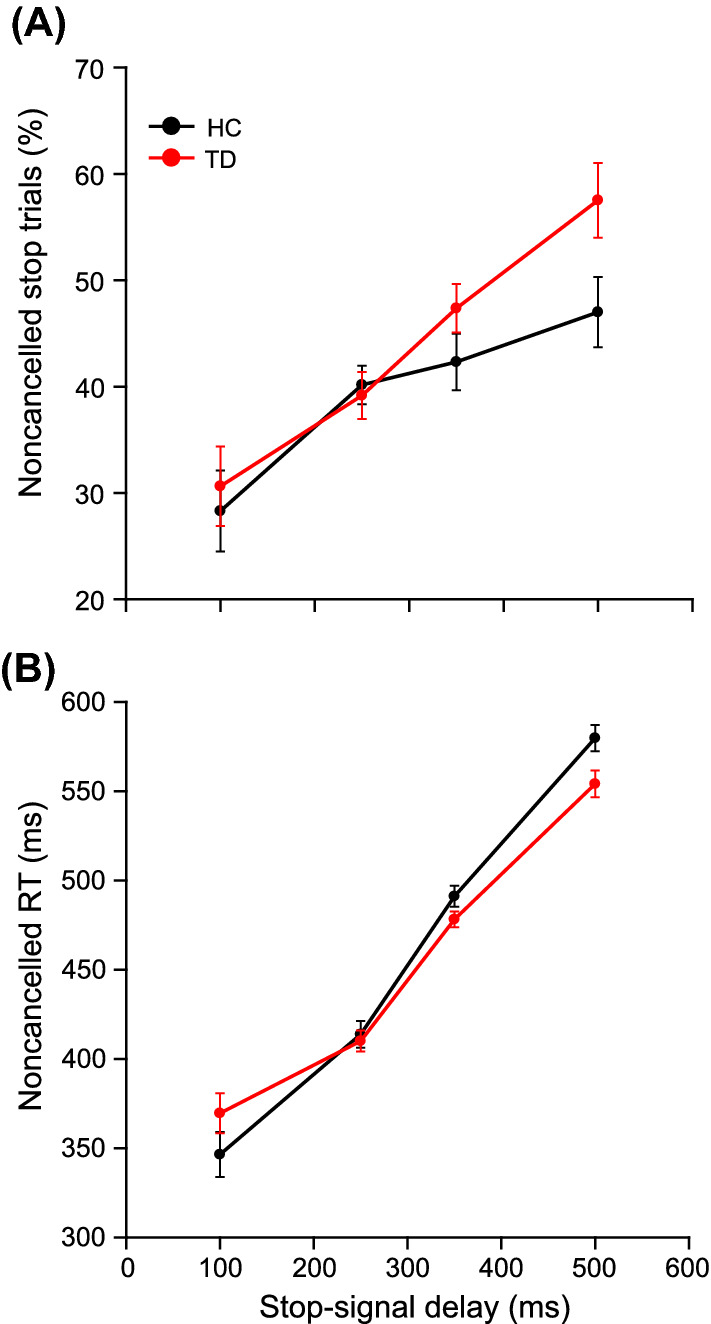
Inhibition function, noncancelled RT as a function of SSD. The SSD was grouped into four (1–200 ms, 201–300 ms, 301–400 ms, and 401–600 ms) bins. The average error in inhibition and noncancelled RT in each bin for every participant was calculated. (**A**) The mean (± SEM) error in inhibition across participants for both groups (HC: black; TD: red) was plotted against the midpoint of the corresponding bin (mean: line; SEM: error bar). The average percentage error in inhibition increased monotonically as a function of SSD in both groups (except a non-significant increase in error from 2nd to 3rd bin in HC). (**B**) The mean (± SEM) noncancelled RT across participants for both groups (HC: black; TD: red) was plotted against the midpoint of the corresponding bin (mean: line; SEM:error bar). The noncancelled RT increased monotonically as a function of SSD in both groups.

No significant differences were found between HC and TD on all behavioral performances (i.e., Go accuracy, error rate, SSD, no-stop RT, and stop error RT; see Table 2). First, we binned the SSD from 0 to 600 ms in the size of 100 ms. Merely 0.004% of total stop trials had SSD greater than 600 ms, which were removed. In the first (1–100 ms) and the last (501—600 ms) bin, merely 3.571% and 28.571% of HC participants and 14.286% and 16.327% of TD participants respectively, contributed trials. As we had no data for a reasonable number of participants, we merged the first two and the last two bins. Thus, bins were 1–200 ms, 201–300 ms, 301–400 ms, and 401–600 ms. We labeled these bins as 1st, 2nd, 3rd, and 4th bins in order. We plotted the mean percentage of noncancelled responses against the midpoint of SSD, each bin for both HC and TD (Fig. 2A). One-way ANOVA with SSD as a repeated factor showed a significant effect of SSD on error in inhibition in both HC [*F* (3,55) = 14.628, *p* < 0.001, ${\upeta }_{\mathrm{p}}^{2}$ = 0.444] and TD group [*F* (3,98) = 47.261, *p* < 0.001, ${\upeta }_{\mathrm{p}}^{2}$ = 0.591]$.$ All pairwise multiple comparison procedures (Holm-Sidak method) showed that mean error significantly increased from 1st to 2nd (*p* = 0.003, *d* = 0.871) and from 3rd to 4th bin (*p* = 0.016, *d* = 0.662) but a non-significant increase from 2nd to 3rd bin (*p* = 0.158, *d* = 0.280) in HC group. Similarly, the mean error increased significantly from 1st to 2nd (*p* < 0.001, *d* = 0.833), from 2nd to 3rd bin (*p* < 0.001, *d* = 0.895), and from 3rd to 4th bin (*p* < 0.001, *d* = 0.726). in the TD group. It shows the error monotonically increased as predicted by the race model in both groups (except error from 2nd to 3rd bin in HC).

**Table 2:** Behavioural performance, SSRT, and CRTT metrics in HC and TD. For both groups, average no-stop accuracy, error in inhibition, stop-signal delay, no-stop RT, and noncancelled RT are shown in the table. None of these parameters was significantly different between HC and TD. SSRT estimated by three methods was not significantly longer in TD than that of in HC group. P-values for difference in mean SSRTs were adjusted by Holm method. Unadjusted p-values were 0.405, 0.64 and 0.664 for integration, median and mean method SSRT respectively. The mean log-attenuation rate was significantly less in TD than that of the HC group, and the mean proactive delay was nearly equal. SSRT was also not significantly different between HC and TD. The standard error of the respective means (± SEM) is given in the brackets.

	Parameters	HC	TD	t	p
Behavioural performance	Go accuracy (%)	97.09 (± 1.20)	98.22(± 0.53)	*t(75)* = *−0.991*	0.325
	Stop error (%)	42.426(± 1.239)	44.414(± 0.817)	*t*(75) = 1.391	0.084
	SSD (ms)	326(± 12)	312(± 10)	*t*(75) = 0.81	0.210
	No stop RT (ms)	556 (± 13)	540 (± 9)	*t*(75) = 1.04	0.302
	Error RT (ms)	476 (± 12)	462 (± 9)	*t*(75) = 0.95	0.346
SSRT	Integration method (ms)	207(± 6)	208 (± 4)	*t*(74) = 0.241	1
	Median method (ms)	227(± 4)	225(± 4)	*t(74)* = *−0.36*	1
	Mean method (ms)	231(± 5)	228(± 4)	*t(75)* = *−0.424*	1
CRTT metrics	Log-attenuation rate	0.0097 (± 0.0003)	0.0088 (± 0.0002)	*t*(75) = *−*2.602	0.006
	Proactive delay (ms)	389 (± 12)	384 (± 9)	*t*(75) = *−*0.342	0.367

### Reaction time

No-stop RT was significantly longer than non-cancelled stop RT in both HC [*t* (27) = 22.03, *p* < 0.001, *d* = 4.164, BF_+0_ > 100] and TD [*t* (48) = 24.39, *p* < 0.001, *d* = 3.485, BF_+0_ > 100]. Subsequently, to test whether noncancelled RT increased with the increase in SSD, we plotted mean noncancelled RT against the midpoint of each SSD bin (Fig. 2B). One-way ANOVA with SSD as repeated factor showed a significant effect of SSD on noncancelled RT in both HC [*F* (3,54) = 129.717, *p* < 0.001, ${\upeta }_{\mathrm{p}}^{2}$ = 0.878] and TD group [*F* (3,95) = 95.29, *p* < 0.001, ${\upeta }_{\mathrm{p}}^{2}$ = 0.751]$.$ Within both groups, mean noncancelled RT was longer than the RT in the preceding bin in all possible pairs (all *ps* <  = 0.009, all *ds* >  = 0.493) reflecting the replication of the predictions of the race model. In short, noncancelled RT increased monotonically as a function of SSD in both groups as predicted by the race model. These results were majorly in line with the race model’s prediction allowing estimation of SSRT.

### Stop-signal response time

We estimated SSRT by three methods to rule out any bias due to the choice of estimation method. Using the three previously described SSRT estimations^3,37,38^ (i.e., by integration, median, and mean), we found no significant difference in average SSRT between TD and HC (Table 2). Subsequently, we checked whether SSRT correlates with overall error in inhibition. In both HC and TD, SSRT estimated by the integration method was significantly correlated with error in inhibition, HC [*r* (25) = 0.632, *p* (one-tailed) < 0.001, *R*^2^ = 0.399, BF_+0_ > 100], TD [*r* (45) = 0.656, *p* (one-tailed) < 0.001, *R*^2^ = 0.430, BF_+0_ > 100]. SSRT estimated by median method had positive significant correlation with error in HC [r (24) = 0.331, p (one-tailed) = 0.049; R^2^ = 0.11, BF_+0_ = 1.673] and non-significant in TD [*r* (46) = 0.003, *p* (one-tailed) = 0.491, *R*^2^ < 0.001, BF_0+_  = 5.46]. SSRT by mean method was also positively correlated in HC [*r* (25) = 0.43, *p* (one-tailed) = 0.026; *R*^2^ = 0.185, BF_+0_ = 5.096] and non-significant in TD [*r* (46) = 0.248, *p* (one-tailed) = 0.088, *R*^2^ = 0.062, BF_+0_ = 1.4]. A significant positive correlation between SSRT and error in inhibition demonstrates that the estimated SSRT was in compliance with race model. However, the model does not show a difference in mean SSRT between TD and HC, suggesting that TD patients do not have compromised inhibitory process.

### Cancellable rise-to-threshold metrics

**Figure 3:**
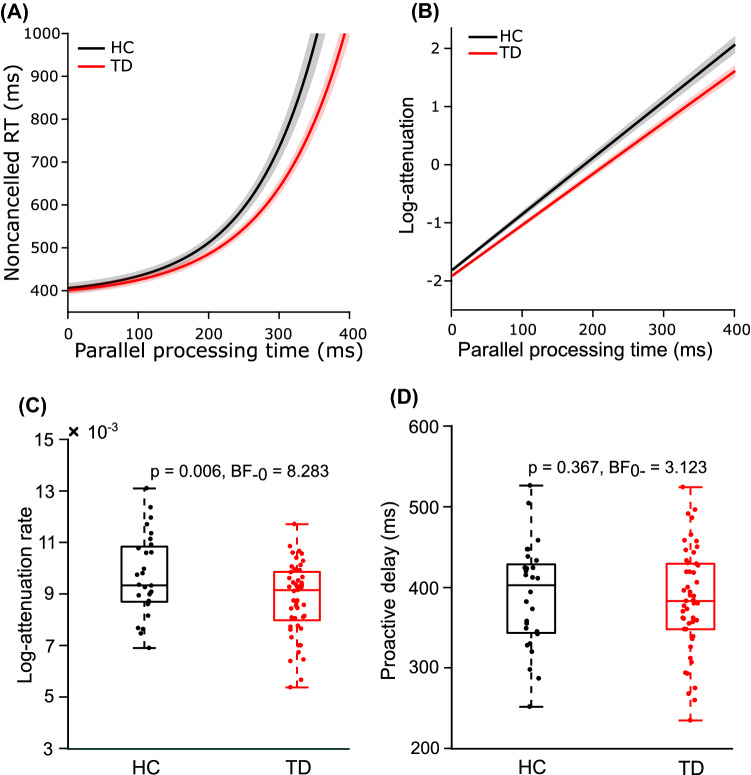
CRTT metrics between TD and HC. (**A**) We fitted noncancelled RT and PPT data with an exponential function ($\mathrm{noncancelled\ RT}=\upvarepsilon {\mathrm{ e}}^{\mathrm{b}(\mathrm{PPT})}+\mathrm{c}$). Coefficient ‘b’ and ‘c’ were fitting coefficients that varied across participants. Constant $\varepsilon$ is a fixed nominal error in the estimation of RT. We fixed ε at 17 ms which was almost equal to one refresh duration of the display monitor to account for random jitter in the measurements of RT and SSD. The Average (± SEM) of best fit across participants in both groups (HC: black; TD: red) are plotted as a function of PPT (average: line; SEM: patch). It shows that there was an overall exponential increase in noncancelled RT in both HC and TD groups. The intercept at the ordinate for each fit in both groups was calculated which mathematically equals $\upvarepsilon +\mathrm{c}$. It is referred to as CRTT metric proactive delay. Since $\upvarepsilon$ was the jitter in the measurement in RT that depended on the refresh rate of the display, c was used as a proactive delay. Mean proactive delay was nearly equal in both groups. (**B**) Log of attenuation was calculated by the equation: $log\left(attenuation\right)=\mathrm{ log}\left(\varepsilon b\right)+ b \times PPT,$ at 1 ms steps from 0 to 400 ms of PPT. The average (± SEM) log-attenuation for both groups (HC: black; TD: red) is plotted as a function of PPT (average: line; SEM: patch). (**C**) The slope of the individual log-attenuation plot is referred to as the log-attenuation rate CRTT metric. Boxplot of distributions of log-attenuation rate for HC (n = 28, black) and TD (n = 49, red) are shown in the figure. The average log-attenuation in the TD group was significantly less than that of the HC group. (**D**) Boxplot of distributions of proactive delay for HC (black) and TD (red) are shown in the figure. There was no significant difference in mean proactive delay between TD and HC.

Using CRTT metrics, we found that the mean log-attenuation rate was significantly less in TD than HC [t (75) = −2.602, *p* (one-tailed) = 0.006, *d* = −0.616] (Fig. 3C). This difference was further supported by Bayesian t-test with ‘substantial’ evidence (BF_-0_ = 8.283). However, no significant difference was observed for the mean proactive delay [t (75) = −0.342, p (one-tailed) = 0.367, d = −0.081] (Fig. 3D), supported by Bayesian t-test with ‘substantial’ evidence (BF_0-_ = 3.123). These results suggest that the TD group had a deficit in reactive control, but proactive control was intact, in comparison to the HC group.

### Correlation of CRTT metrics with error in inhibition

Theoretically, log-attenuation rate and proactive delay, each should have a negative correlation with the overall error in inhibition. We tested the correlation of log-attenuation rate and proactive delay with overall error in inhibition for both groups. Log-attenuation rate was significantly correlated with error in both HC [*r* (25) = −0.514, *p* (one-tailed) = 0.003, *R*^2^ = 0.264, BF_-0_ = 16.183] and TD [*r* (44) = −0.247, *p* (one-tailed) = 0.049, *R*^2^ = 0.058, BF_-0_ = 1.310]. However, Bayesian statistics suggests this relationship was ‘anecdotal’ (1 ≤ BF < 3) for TD, but ‘strong’ (10 ≤ BF < 100) for HC. Pearson’s coefficient indicates that the magnitude of coupling between log-attenuation rate and error in HC was almost numerically twice the magnitude of coupling in TD. However, a follow-up Fisher’s z test estimated by cocor package^39^ showed that the magnitude of correlations of log-attenuation rate and error between HC and TD was not significantly different [z = −1.240, *p* (two-tailed) = 0.215]. Proactive delay was also significantly correlated with error in both HC [*r* (25) = −0.775, *p* (one-tailed) < 0.001, *R*^2^ = 0.601, BF_-0_ > 100] and TD [*r* (44) = −0.735, *p* (one-tailed) < 0.001, *R*^2^ = 0.540, BF_-0_ > 100]. Bayesian statistics suggests this relationship was ‘decisive’ (BF ≥ 100) for both groups.

### Influence of ADHD and medication on CRTT metrics

Since we found a link between deceleration rate and attention in Indrajeet and Ray (2019) and effect of medication on SSRT in Atkinson-Clement et al*.* (2020), we subsequently verified whether presence of ADHD or medication influenced CRTT metrics (see Table 3). We made the following observations: (1) The mean (± SEM) log-attenuation rate in TD with ADHD (0.0087 ± 0.0004) and TD without ADHD (0.0089 ± 0.0002) was not significantly different [*t* (47) = −0.364, *p* (two-tailed) = 0.717, *d* = −0.106, BF_01_ = 3.279]. (2) The mean (± SEM) proactive delay in TD with ADHD (384 ± 15 ms) and TD without ADHD (384 ± 12 ms) was not significantly different [*t* (47) = 0.003, *p* (two-tailed) = 0.997, *d* < 0.001, BF_01_ = 3.461]. (3) The mean (± SEM) inhibition error in TD with ADHD (44.07 ± 1.42%) and TD without ADHD (44.65 ± 0.99%) was not significantly different [*t* (47) = −0.343, *p* (two-tailed) = 0.733, *d* = −0.1, BF_01_ = 3.299]. (4) The mean (± SEM) log-attenuation rate in medicated TD (0.0086 ± 0.0003) and un-medicated TD (0.0089 ± 0.0003) was not significantly different [*t* (47) = −0.828, *p* (two-tailed) = 0.412, *d* = −0.243, BF_01_ = 2.601]. (5) The mean (± SEM) proactive delay in medicated TD (366 ± 16 ms) and un-medicated TD (395 ± 11 ms) was not significantly different [*t* (47) = −1.596, *p* (two-tailed) = 0.117, *d* = −0.468, BF_01_ = 1.235]. (6) The mean (± SEM) inhibition error in medicated TD (45.89 ± 1.51%) and un-medicated TD (43.48 ± 0.91%) was not significantly different [*t* (47) = 1.452, *p* (two- tailed) = 0.153, *d* = 0.426, BF_01_ = 1.471]. These results indicate that the difference found in log-attenuation rate and proactive delay in HC and TD might not be due to medication or presence of ADHD. We did not consider OCD due to small sample size (n = 11) and lack of prior link between CRTT metrics and OCD.

**Table 3:** Relationship of ADHD and medication with CRTT metrics and stopping error. The TD group was divided into subgroups: (1) TD with ADHD vs TD without ADHD, and (2) TD with medication vs TD without medication. Table shows that both CRTT metric and average error in inhibition was not significantly different between sub-group comparisons 1 and 2.

	Log-attenuation rate (mean ± SEM)	Proactive delay (ms) (mean ± SEM)	Stop error (%) (mean ± SEM)
TD with ADHD	0.0087 ± 0.0004	384 ± 15	44.07 ± 1.42
TD without ADHD	0.0089 ± 0.0002	384 ± 12	44.65 ± 0.99
p	0.717	0.997	0.733
BF_01_	3.279	3.461	3.299
Medicated TD	0.0086 ± 0.0003	366 ± 16	45.89 ± 1.51
Un-medicated TD	0.0089 ± 0.0003	395 ± 11	43.48 ± 0.91
p	0.412	0.117	0.153
BF_01_	2.601	1.235	1.471

## Discussion

In this study, we applied both traditional race model^3^ and relatively recent CRTT model^9^ to assess the inhibitory control in the TD group in comparison to HC participants. Not surprisingly, like earlier studies we also observed that neither stopping behavior nor the metric derived from race model was able to distinguish between TD and HC. On the contrary, our new method based on CRTT model teased apart contributions from proactive and reactive control in stopping a manual movement. While the ability to postpone an action by TD was comparable to HC, the log-attenuation rate that estimates the strength of deceleration in GO process following the stop signal was significantly less in TD than HC. Our study also suggests that the CRTT metrics, which were originally derived to study countermanding saccades, can be generalized to variants of stop-signal task with different effectors (eye / hand) and stop-signal adjustment methods (randomization/staircasing). These results are consistent with a recent meta-analysis study indicating possible impairment in reactive but not in proactive inhibitory control in TD^40^, and critical from the clinical point of view to identify differences in neural circuitry causing deficiency in one type of control sparing the other.

What might be a feasible physiological explanation of weak attenuation on GO process, but intact ability of strategic adjustment of the time of process initiation (postpone the movement) in the TD group? Studies have advocated that the strength of the fronto-basal ganglia connectivity largely determines the efficacy of the inhibitory control^41–51^ and dysfunctional cortico-basal ganglia pathways underlie TD^21,52–55^. Interestingly, inhibition of preplanned action entails an engagement of fronto-cortical basal ganglia pathways^43,46–51^, which is somehow compromised in TD^21,54,55^. A simplified description suggests that on the direct pathway in basal ganglia, the dorsal striatum that receives projection from the frontal cortex inhibits the substantia nigra pars reticulata (SNr) causing disinhibition of the thalamus, which is attributed to an underlying neural mechanism of the GO process, whereas the subthalamic nucleus (STN) exerts an excitatory influence on SNr acting similar to pressing the brake in a car^43,51,56,57^. The dorsal striatum consists of two structures: caudate nucleus (CN) and putamen (Pu). Striatum and STN are also interconnected via globus pallidus external (GPe) on the indirect pathway^51,58,59^. Imaging studies have found an abnormality in cortico-basal ganglia-thalamocortical motor loop and supplementary motor area in TD^20,60^. TD patients exhibited increased activation in the right supplementary motor area and the inferior parietal cortex, but decreased activation in the dorsal pre-motor cortex while performing a stop-signal task^20,61^. Furthermore, Ganos et al. (2014) showed that the activation of supplementary motor area (SMA) on correct stop trials were correlated with the frequency of tics.

Similarly, some EEG studies have shown that the amplitude of fronto-central P300, considered as a marker of response inhibition^62–64^, was diminished in TD patients in comparison to HC^65^. However, P300 as a marker of response inhibition is debated^66^ and may reflect other processes e.g., infrequent stimulus detection as well^67,68^. Additionally, EEG studies in TD (in a different context than stop-signal task) doesn’t consistently show diminished P300^69^ and it is not always attributed to impaired inhibitory process. For example, reduced amplitude of P3b in TD was linked to altered allocation of attentional resources^70^. Some studies have shown that P300 linked to no-go is shifted over anterior (frontal) from central electrodes in TD^71,72^ but no difference in P300 amplitude between TD and HC^72^, and even enhance P3b amplitude at parietal electrodes^73^ or delayed P300 onset in TD in incompatible condition in Simon task^74^. Error related negativity amplitudes were not significantly different between TD and HC implying intact error detection^75,76^ (for a review of ERPs in TD refer to Morand-Beaulieu and Lavoie 2019^69^). Since we have previously shown a link between detectability of stop-signal and attenuation rate^32^, it may be an interesting idea to explore the relationship between ERP components (e.g., P300) and CRTT metrics in future. Interestingly, despite the differential neural activity in brain areas critical for response inhibition, none of these studies found a significant difference in SSRT or other behavioural performance between TD and HC. At the structural level, the volume of the caudate nucleus (CN), putamen (Pu), and globus pallidus (GPe) are reduced in TD adults^55,77,78^. Single neuron’s activity recordings have shown a link between neuronal activity in the caudate nucleus in monkeys^79^ and GPe neurons in rats^51,56^ in reactive inhibition. GPe neurons activity in rats is also linked to proactive control during stop-signal tasks^80^. We speculate that SSRT is not always a reliable metric to identify some of the differences found at the neural level in TD that might be explained by CRTT metrics.

Our study suffers from a few limitations. For example, CRTT metrics could not be computed for each emotion category and for each participant due to a smaller number of noncancelled trials in each emotion condition. It would be interesting to test whether CRTT metrics are sensitive enough to detect modulation of the inhibitory process in an emotional context and able to resolve the contradictory findings regarding the effect of emotion on inhibitory control. In addition, some recent studies showed that the neurocognitive measures of inhibitory control were not always the best predictors of tics severity as did the subjective tics suppressibility^81^. Also, presence, severity, and capacity to inhibit premonitory urges could be relevant to the present results^82,83^. Further studies are warranted to address these caveats. Recent studies have shown impairment in inhibition in TD is not purely motor but also linked to perceptual and cognitive processes, e.g., altered perception–action integration^84,85^, attention^86^, etc. (for a viewpoint refer to review^87^). Additional studies which measure perceptual and cognitive processes with the inhibition are warranted to show a wholistic view of the nature of impairment in TD. Further, like many other clinical sample studies, comorbidities and medication might be issues. However, we found that CRTT metrics were not different between TD with and without ADHD, or un-medicated and medicated TD. Having said that caution should be taken interpreting no statistical difference, because effects were analyzed separately ignoring the effect of other factors. Despite these limitations, our study is a methodological advancement in the assessment of deficits in inhibitory control in TD.

## Materials and methods

### Participants

A total of 63 TD and 34 HC were recruited for the original project. The HC group was matched with the TD group for age, gender, and acquired education, keeping the ratio between the healthy control and TD patients ~ 1:2. The exclusion criteria for all participants were based on satisfying any of the following conditions: (1) incapable to provide consent, (2) history of substance (except nicotine) addiction or psychosis, (3) neurological condition except tics, (4) history of any psychiatric condition for HC. All participants were informed about the procedure and protocol of the study and gave their informed consent. Monetary compensation (50 Euros) was provided for participation.

### Ethics

The study was approved by the Comité de protection des personnes (CCP) of Sorbonne Université (CCP16163/C16-07) and preregistered prior to the research being conducted on ClinicalTrial (https://clinicaltrials.gov/show/NCT02960698). All participants gave their informed consent. All procedures comply with the ethical standards of the relevant national and institutional committees on human experimentation and with the Helsinki Declaration of 1975, as revised in 2008.

### Clinical measures

All participants were assessed using the Mini-International Neuropsychiatric Interview (MINI)^88^ for psychiatric disorders, the Minnesota Impulse Disorders Interview (MIDI)^89^ for impulse-control related disorders, and the Barratt Impulsivity Scale (BIS)-11^90^ for impulsivity. For TD patients, the Yale Global Tic Severity Scale (YGTSS)^91^ was used to assess the severity of tics. Each patient underwent a psychiatric evaluation and a medical history check-up to assess for psychiatric comorbidities.

### Stimuli and task

As shown in Fig. 1, each trial began with a white fixation cross for 500 ms at the centre of the display. It was followed by an image of a scene with emotional content for the duration of 700 ms at the centre. A total of 135 images^92^ were selected from the international affective picture system and were from three groups of equal size ( neutral, positive and, negative). Positive or negative emotional content images were of moderate emotional intensity and arousal. Thus, in a total of 405 trials, each image was presented thrice. However, we could not fit the CRTT model for each emotion condition due to insufficient number of error stop trials with positive PPT. Therefore, all trials in each emotion condition were combined for this study. After a blank black display for a 1–2 s from the image, letter ‘O’ in green appeared at the centre of black display acting as a go-signal (go-trials). Participants were instructed to press the ENTER key ‘as soon as possible’ in these trials. However, in 33% of the total trials, after a variable delay from the go-signal (stop-signal delay), letter ‘X’ painted in red appeared at the centre acting as a stop-signal (stop-trials). Participants were instructed to refrain from pressing the Enter key in these trials. For a detailed description of the task e.g., staircasing of SSD, refer to supplementary material. Details of scene image selection and categorization, and inhibitory control in induced emotional context can be found elsewhere^21^.

### Apparatus and procedure

E-prime software (Psychology Software Tools, Pittsburgh, PA) created and displayed all stimuli and stored the critical task parameters and responses made by participants. All stimuli were displayed on a monitor at a 60 Hz refresh rate. The ENTER key of the keyboard was assigned for response input. The pre-processing and basic analysis (e.g., descriptive statistics, estimation of SSRTs) of the data was carried out by using Matlab® (The Mathworks Inc., USA). Matlab® Statistical Toolbox (The Mathworks Inc., USA) (correlations, t-tests), JASP software (JASP team, 2019, version 0.11.1) (Bayesian statistics) and SigmaStat (SYSTAT Inc., USA, version 4.0) (ANOVAs and post-hoc tests) was used for statistical analysis. Curve Fitting Toolbox™ of Matlab® was used for fitting curves.

### Estimation of CRTT metrics

The CRTT model advocated that the preparatory build-up activity is decelerated after the stop-signal is detected if and only if the stop-signal is detected in a stipulated time and the magnitude of deceleration is sufficient to prevent the preparatory activity building up over time from reaching a threshold^36^. We previously extended the CRTT model to show an exponential increase in reaction time (RT) in unsuccessful stop trials, as parallel processing time (PPT) increased^32^. PPT is defined as the maximum time duration, for which the stop-signal and the go signal can be processed in parallel before the elicitation of a response. Empirically, it was calculated by subtracting SSD from RT in error stop trials (i.e., noncancelled RT–SSD). PPT was grouped in bins and mean PPT and corresponding mean noncancelled RT was calculated for each bin. Noncancelled RT was plotted against PPT, and the plot was fitted with an exponential function ($\mathrm{noncancelled RT}=\upvarepsilon {\mathrm{ e}}^{\mathrm{b}(\mathrm{PPT})}+\mathrm{c}$) (Fig. 3A). The rate of log of the slope of the best exponential fit is referred to as log-attenuation rate (Fig. 3B), and the intercept of the best exponential fit is referred to as proactive delay. For a detailed derivation of CRTT metrics, refer to supplementary material or our previous work^9^.

### Statistical analyses

The criterion of significance α was fixed at 0.05 level, except for the tests of normality and equivalence of variance where a lenient criterion was used (α = 0.01). Linear Pearson product–moment correlation method was performed to calculate correlations (see supplementary materials for more details). SSRT was estimated by three methods^3,37,38^ (i.e., by integration, median, and mean) for HC and TD groups. P-values of t-tests (to compare mean SSRT between HC and TD) and correlations (between SSRT and error in inhibition) were adjusted for multiple comparisons using Holm method^93^ using R software (R Core Team, 2020, version 4.1.1).

## Data availability

Data may be shared in its raw form for review purpose or other reasonable scholarly use.

## Supplementary Information

Supplementary Information.
