# Supplementary material for: Compromised reactive but intact proactive inhibitory motor control in Tourette disorder
Source: Sci Rep. 2022 Feb 9;12:2193. doi: 10.1038/s41598-022-05692-z (PMC8828748; doi:10.1038/s41598-022-05692-z)
Supplement: Supplementary file 1 — Supplementary Information. [file 41598_2022_5692_MOESM1_ESM.pdf]

Supplementary Materials for

Compromised reactive but intact proactive inhibitory motor control in Tourette disorder

Indrajeet Indrajeet, Cyril Atkinson-Clement, Yulia Worbe, Pierre Pouget, Supriya Ray

Email: pierre.pouget@upmc.fr (PP); sray@cbcs.ac.in (SR)

This PDF file includes:

Supplementary Text

Subheadings:

1. **Emotional Stop Signal Task**
2. **CRTT metrics**
3. **Statistics**
4. **CRTT model fitting and outlier criteria**

SUPPLEMENTARY TEXT

Emotional Stop Signal Task (ESST)

After a variable interval between 1-2 s from the image, the letter O in upper case and painted in green color appeared at the center of the black screen, which acted as a *go-signal* instructing the participant to press the ENTER key of the keyboard *"as soon as possible". Maximum* allowed period to press the key was 1000 ms, otherwise, the trial was aborted. The duration between the onset of the go-signal and the keypress is referred to as go (or no-stop) reaction time. In 33 % of the total 405 trials, called *stop trials*, after a variable delay from the onset of the circle, the letter X in upper case and painted in red color replaced the letter O at the center. The appearance of the letter X acted as the *stop-signal* instructing participants to refrain from pressing the key. The delay between onset of the go- and stop-signal is called stop-signal delay (SSD), and it was adjusted by the tracking procedure. In this procedure, initially, the SSD was fixed at 250 ms in each emotion category. Subsequently, SSD was increased by 25 ms, if the previous stop trial was correct (i.e., the response was cancelled); otherwise, SSD was decreased by 25 ms. In the rest of 66 % of trials, called *go-trials* or *no-stop trials*, the stop-signal did not appear, and participants were required to press the ENTER key. No-stop and stop trials were randomly interleaved. No feedback about the outcome (correct or incorrect) of a trial was provided. However, if the go response was too slow (> 1000 ms), a message requesting to generate a faster response in the next trials was displayed.

CRTT metrics

We defined PPT as the maximum time duration, for which the stop-signal and the go signal were processed in parallel before the elicitation of a response. Empirically, it was calculated by subtracting SSD from RT in error stop trials (i.e., noncancelled RT – SSD). When the stop-signal appeared after the response was generated (PPT<0), it could not influence the ongoing motor plan. Therefore, only a subset of trials that yielded PPT ≥0 was used for the estimation of CRTT metrics. We estimated these metrics by fitting noncancelled RT and PPT data with an exponential function ($\text{noncancelled RT} = \varepsilon\, e^{b(\text{PPT})} + c$). We previously derived CRTT metrics to assess the efficacy of inhibitory control in HC[9]. In brief, the rate of change of non-canceled RT

concerning PPT gives the slope of the function $\mathrm{RT} = \varepsilon\, \mathrm{e}^{\mathrm{b(PPT)}} + \mathrm{c}$, which estimates attenuation exerted on GO process building up over time to reach a decision threshold as a function of PPT [$attenuation = \varepsilon \mathrm{b}\, \mathrm{e}^{\mathrm{b(PPT)}}$]. After taking the natural log of both sides we get, $\ln(attenuation) = \ln(\varepsilon b) + b \times PPT$. Alternatively, $\ln(attenuation) = b \times PPT + b_0$, where b_0 equals ln (εb). Given that *b* is obtained from the fitting algorithm and fixed for each participant, and ε is a fixed nominal error in the estimation of RT, the rate of change in attenuation in log-scale with respect to PPT equals to b. We refer to this fitting coefficient as 'log-attenuation rate', which is one of the CRTT metrics. Whereas the other CRTT metric, which we refer to as 'proactively delay', estimates participant's ability to procrastinate response elicitation in anticipation of the stop-signal. Proactive delay is calculated by inserting PPT= 0 in $\mathrm{RT} = \varepsilon\, \mathrm{e}^{\mathrm{b(PPT)}} + \mathrm{c}$. Since ε is constant and nominal in comparison to noncancelled RT, $(\varepsilon + \mathrm{c})$ is approximated to the fitting coefficient *c* to estimate proactive delay.

Statistics

We first compared behavioral performance (e.g., average error and RT in go and stop trials) in stop-signal tasks and between HC and TD using t-tests. We used one-way repeated measures ANOVA to see whether stop error and RT in error stop trials increased with SSD. Holm-Sidak method was used for all pairwise comparisons and effect-size Cohens' d was calculated wherever required. Subsequently, we compared SSRT between TD and HC estimated by three methods by t-tests and checked the correlation of SSRT with average error in inhibition. Next, we fitted the CRTT model to the plots of noncancelled RT against PPT for both groups. Bayesian versions of tests with recommended (e.g., default prior) settings in JASP were performed and Bayes factor (BF) is reported that was specifically aimed for null results' interpretation. With the JASP default value of the r-factor (0.707), we fixed criteria of BF values for classifying strength as anecdotal ($1 \le BF < 3$), substantial: $3 \le BF < 10$, strong: $10 \le BF < 100$, and decisive: $BF \ge 100$. JASP denotes non-directional null and alternate hypotheses as 0 and 1 respectively that are incorporated as BF_{01} (evidence in favour of null) and BF_{10} (evidence in favour of alternate). For directional hypotheses, digit 1 is replaced by + or – sign; for example, BF for positive and negative correlations are denoted by BF_{+0} (evidence for positive correlation i.e., alternate hypothesis), BF_{-0} (evidence for negative

correlation i.e., alternate hypothesis) respectively.

CRTT model fitting and outlier criteria

Since we did not have a reasonable amount of data for each emotion category for CRTT model fitting, we could not take emotion into account. We grouped noncancelled stop trials of each participant from 0 ms to 400 ms in bins of size 80 ms. The mean PPT and mean noncancelled RT were calculated across trials in each bin. The mean noncancelled RT was plotted against the mean PPT for each participant and fitted with the exponential function ($\text{noncancelled RT} = \varepsilon\, e^{b(PPT)} + c$). We fixed ε at 17 ms which was almost equal to one refresh duration of the display monitor to account for random jitter in the measurements of RT and SSD. In the HC, one participant had data only in two bins of PPT, which was less than the minimum data points required to fit it to the exponential function, so the participants' data were removed. The goodness-of-fit for two participants, one in each group (HC: $R^2 = 0.26$, TD: $R^2 = 0.001$), were less than the minimum fixed criterion ($R^2 < 0.5$). Besides, we used the box-and-whisker plot method or Tukey method to detect univariate outliers, if any, in b or c in both groups. Three participants from the TD group were marked outlier by the box-plot method (b values: 0.0130, 0.0129, and 0.0130, number of IQR from median: 1.9979, 1.93, and 1.978). No univariate outliers were detected in c in the TD group. No outliers were detected in b or c in the HC group. Thus, two participants' data from HC and four from the TD group were removed as outliers from all subsequent analyses. *The final sample contains 28 HC and 49 TD.* Besides, in the HC group, one participant had an SSRT of 19 ms, which is theoretically not possible, so it was removed from wherever SSRT of the HC was used. In correlation analyses, scatter plots with fitted regression lines with 95 % confidence bound boundaries were used. Any data points falling out of the prediction bound were removed as outliers from the correlation analysis.
